# Draft genome sequence of *Acidithiobacillus thiooxidans* CLST isolated from the acidic hypersaline Gorbea salt flat in northern Chile

**DOI:** 10.1186/s40793-017-0305-8

**Published:** 2017-12-19

**Authors:** Raquel Quatrini, Lorena V. Escudero, Ana Moya-Beltrán, Pedro A. Galleguillos, Francisco Issotta, Mauricio Acosta, Juan Pablo Cárdenas, Harold Nuñez, Karina Salinas, David S. Holmes, Cecilia Demergasso

**Affiliations:** 1 Fundación Ciencia & Vida, Av. Zañartu 1482, 7780272 Santiago, Chile; 2 Centro de Biotecnología “Profesor Alberto Ruiz”, Universidad Católica del Norte, 1270709 Antofagasta, Chile; 3 Centro de Investigación Científica y Tecnológica para la Minería, Antofagasta, Chile; 4 uBiome, Inc., San Francisco, California, USA; 5 Compañía Minera Zaldívar, Antofagasta, Chile; 6 Facultad de Ciencias Biológicas, Universidad Andrés Bello, Santiago, Chile

**Keywords:** *Acidithiobacillaceae*, Halotolerance, Osmotolerance, Sulfur oxidization, Flexible gene complement, Bioleaching, Mars analog, Salar de Gorbea

## Abstract

10.1601/nm.2199 CLST is an extremely acidophilic gamma-proteobacteria that was isolated from the Gorbea salt flat, an acidic hypersaline environment in northern Chile. This kind of environment is considered a terrestrial analog of ancient Martian terrains and a source of new material for biotechnological applications. 10.1601/nm.2199 plays a key role in industrial bioleaching; it has the capacity of generating and maintaining acidic conditions by producing sulfuric acid and it can also remove sulfur layers from the surface of minerals, which are detrimental for their dissolution. CLST is a strain of 10.1601/nm.2199 able to tolerate moderate chloride concentrations (up to 15 g L^−1^ Cl^−^), a feature that is quite unusual in extreme acidophilic microorganisms. Basic microbiological features and genomic properties of this biotechnologically relevant strain are described in this work. The 3,974,949 bp draft genome is arranged into 40 scaffolds of 389 contigs containing 3866 protein-coding genes and 75 RNAs encoding genes. This is the first draft genome of a halotolerant 10.1601/nm.2199 strain. The release of the genome sequence of this strain improves representation of these extreme acidophilic Gram negative bacteria in public databases and strengthens the framework for further investigation of the physiological diversity and ecological function of 10.1601/nm.2199 populations.

**Electronic supplementary material:**

The online version of this article (10.1186/s40793-017-0305-8) contains supplementary material, which is available to authorized users.

## Introduction

The genus 10.1601/nm.2198 comprises a group of obligatory acidophilic, Gram negative, rod shaped bacteria that derive energy from the aerobic oxidation of reduced sulfur compounds (RISCs) to support autotrophic growth. In the process of oxidizing RISCs, these bacteria produce sulfuric acid and contribute to the bioleaching of ores. Currently, the genus comprises seven described species, 10.1601/nm.2199 ATCC 19377, 10.1601/nm.2202 ATCC2327*,* 10.1601/nm.2200 ATCC35403**,** 10.1601/nm.2201 DSM 8584 [1], 10.1601/nm.17776 [2], *Acidtithiobacillus ferridurans* [3] and 10.1601/nm.27980 [4]. Despite being the first acidophile ever isolated [5], 10.1601/nm.2199 investigation lags behind other members of the genus, especially when compared to the iron oxidizer 10.1601/nm.2202, for which extensive knowledge on its basic ecophysiology and biotechnological use has been gathered [6].

The draft genomes of ten isolates of 10.1601/nm.2199 are available: the type strain 10.1601/strainfinder?urlappend=%3Fid%3DATCC+19377 obtained from the Kimmeridge clay formation in England [7], the strain DSM 17318 named Licanantay isolated from a copper mine in northern Chile [8], the A01 strain isolated from wastewater of a coal dump in China [9] and seven other isolates obtained from copper mines (BY-02, DXS-W, GD1-3, TYC-17, ZBY) and coal heaps (A02, DMC) in China [10].

The 10.1601/nm.2199 type strain (10.1601/strainfinder?urlappend=%3Fid%3DATCC+19377) is motile, grows on elemental sulfur, thiosulfate or tetrathionate, and has temperature optimum of 30 °C and a pH optimum of 2.0 to 3.0 [1]. Members of the species have been found to occur in a variety of natural-acidic and man-made environments, including sulfidic caves [11], shales [12], fresh water [13], sea water [14], sewer pipes [15], mineral leaching heaps [16], mine dumps [17] and mine wastes [18] from different parts of the world. With the exception of 10.1601/nm.2199 strain SH isolated from sea water, which has a confirmed requirement of NaCl (2%; 0.35 M) for growth in synthetic media [14], all characterized 10.1601/nm.2199 strains are inhibited by even moderate NaCl concentrations [19].

10.1601/nm.2199 CLST is a new NaCl tolerant strain (15 g L^−1^ Cl^−^) isolated from the Gorbea salt flat in the Central Andean plateau (Bolivia, Chile and Argentina, between 19° and 27° S latitude). This salt flat is located in an endorheic basin displaying strongly acidic brines (with a pH between 2 and 4 and a salinity ranging between 1.7 - 76.9 g L^−1^ NaCl) and one of the few acid saline systems known worldwide [20–22]. These uncommon types of natural extreme environments are considered terrestrial analogs to certain ancient Martian terrains and a source of new material for biotechnological applications [23, 24].

This work reports the microbiological properties of this NaCl-tolerant acidophilic sulfur-oxidizing 10.1601/nm.2198 from the saline environment in northern Chile, together with its draft genomic sequence and annotation. The release of the genome of the CLST strain will contribute to a better understanding of the ecophysiology of extreme acidophiles inhabiting saline environments and of sodium-requiring processes (e.g. symport, antiport, flagellar rotation, etc.), in acidophilic chemolithotrophic bacteria. Knowledge derived from the study may also provide new opportunities in biotechnological and astrobiological endeavors.

## Organism information

### Classification and features

**Fig. 1:**
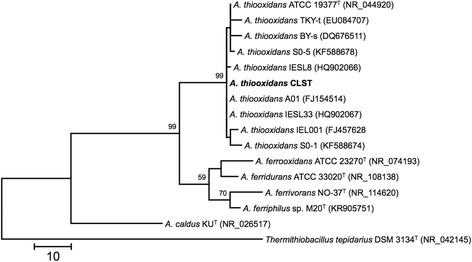
Phylogenetic tree based on the 16S rRNA gene sequences highlighting the position of 10.1601/nm.2199 strain CLST relative to other type and non-type strains of the genus 10.1601/nm.2198. The GenBank database accession codes are indicated between brackets. The evolutionary history was inferred by using the Maximum Parsimony and the Subtree-Pruning-Regrafting (SPR) algorithm with search level 1 [52]. The initial trees were obtained by the random addition of sequences. The analysis involved 16 nucleotide sequences and a total of 1307 non-ambiguous positions in the final dataset. Evolutionary analyses were conducted in MEGA version 6.22 [53]. Tree construction used a bootstrapping process repeated 1000 times to generate a majority consensus tree. A sequence from 10.1601/nm.2205 was used as outgroup. The tree is drawn to scale, with branch lengths calculated using the average pathway method [53]; the scale bar corresponds to the number of changes over the whole sequence

**Fig. 2:**
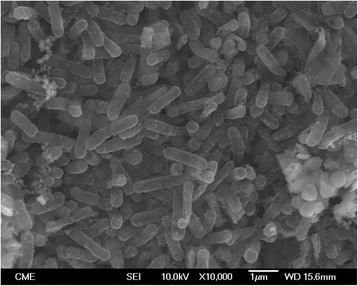
Scanning electron microscopy image of 10.1601/nm.2199 strain CLST

10.1601/nm.2199 CLST was isolated at the Biotechnology Center (CBAR-UCN) from a sulfur enrichment culture designed to select acidophilic bacteria that could oxidize RISCs under saline conditions. Briefly, salt-water samples obtained from the Gorbea salt flat were inoculated in a batch reactor containing minimal medium [25] and elemental sulfur as energy source. Phylogenetic analysis of the 16S rRNA sequence indicated that the CLST strain (10.1601/strainfinder?urlappend=%3Fid%3DDSM+103717) is related to 10.1601/nm.2199 (Fig. 1)*.* CLST cells are Gram-negative, rod-shaped (0.4 μm × 1-1.5 μm) and motile (Fig. 2). Optimal growth occurs at 28 °C and pH 1.7. It grows autotrophically using sulfur as electron donor and oxygen as the electron acceptor. It is also a facultative anaerobe capable of using RISCs as electron donors and ferric iron as an electron acceptor. Strain CLST forms small white colonies when grown autotrophically on solid medium containing RISCs. It differs from closely related strains, Licanantay and A01 (JMEB00000000 and FJ154514, respectively), in its capacity to grow in 15 g L^−1^ of chloride. The microorganism information is presented in Table 1.

**Table 1:** Classification and general features of 10.1601/nm.2199 CLST

MIGS ID	Property	Term	Evidence code^a^
	Classification	Domain *Bacteria*	TAS [44]
		Phylum 10.1601/nm.808	TAS [45]
		Class 10.1601/nm.24436	TAS [46]
		Order 10.1601/nm.2196	TAS [38]
		Family 10.1601/nm.2197	TAS [39, 47]
		Genus 10.1601/nm.2198	TAS [1]
		Species 10.1601/nm.2199	TAS [1, 5]
		Strain: CLST (10.1601/strainfinder?urlappend=%3Fid%3DDSM+103717)	IDA
	Gram stain	Negative	IDA
	Cell shape	Rod	IDA
	Motility	Motile	IDA
	Sporulation	Not reported	IDA
	Temperature range	25 -35 °C	IDA
	Optimum temperature	28 °C	IDA
	Optimum pH	1.7	IDA
	Carbon source	CO_2_	TAS [25]
MIGS-6	Habitat	Brine, acidic hypersaline environment	IDA
MIGS-6.3	Salinity	10 -15 gL^−1^ chloride	IDA
MIGS-22	Oxygen requirement	Aerobic and facultative anaerobic	IDA
MIGS-15	Biotic relationship	Free-living	TAS [48]
MIGS-14	Pathogenicity	Non-pathogen	NAS
MIGS-4	Geographic location	Gorbea salt flat, Antofagasta region, Chile	IDA
MIGS-5	Sample collection	11/20/2007	IDA
MIGS-4.1	Latitude	25°25′72.2´´S	IDA
MIGS-4.2	Longitude	68°41′53.2´´W	IDA
MIGS-4.4	Altitude	4000 m.a.s.l.	IDA

^a^Evidence codes - *IDA* Inferred from Direct Assay, *TAS* Traceable Author Statement (i.e., a direct report exists in the literature), *NAS* Non-traceable Author Statement (i.e., not directly observed for the living, isolated sample, but based on a generally accepted property for the species, or anecdotal evidence). These evidence codes are from the Gene Ontology project [49]. Data is in compliance with MIGS version 2.0 [50] and the NamesforLife database [51]

## Extended feature descriptions

The growth rate of 10.1601/nm.2199 type strain 10.1601/strainfinder?urlappend=%3Fid%3DATCC+19377 undergoes a significant decrease (μ from 0.76 to 0.52 day^−1^) at NaCl concentration of 325 mM compared with growth on culture medium without the salt (Additional file 1: Figure S1). Meanwhile there is not a significant change in the growth rate of 10.1601/nm.2199 CLST in the same conditions. In addition 10.1601/nm.2199 CLST precipitates CuS when it is grown aerobically in culture medium amended with CuSO_4_ (Additional file 2: Figure S2). This feature has been already observed in *E. coli* associated to the heterologous expression of the enzyme cysteine desulfhydrase [26]. We identified the gene for a previously described cysteine desulfhydrase (CdsH) in the genome of 10.1601/nm.2199 CLST strain. CdsH appears to be the major cysteine-degrading and sulfide-producing enzyme aerobically but not anaerobically [27].

## Genome sequencing information

### Genome project history

The organism was selected for sequencing on the basis of its phylogenetic position and 16S rRNA similarity to members of the genus 10.1601/nm.2198, and for its atypical origin; coming from an extreme acidic and saline biotope. This Whole Genome Shotgun project has been deposited at GenBank under the accession number LGYM00000000. The version described in this paper is the first version, LGYM00000000. The project information is presented in Table 2.

**Table 2:** Project information

MIGS ID	Property	Term
MIGS 31	Finishing quality	Draft
MIGS-28	Libraries used	GS FLX Titanium paired end libraries
MIGS 29	Sequencing platforms	Roche 454 GS FLX
MIGS 31.2	Fold coverage	36 ×
MIGS 30	Assemblers	Newbler 2.0.01.14
MIGS 32	Gene calling method	Glimmer 3.02
	Genbank ID	LGYM00000000
	GenBank Date of Release	2017-04-05
	GOLD ID	Gp0136483
	BIOPROJECT	PRJNA291500
MIGS 13	Source Material Identifier	Gorbea-A
	Project relevance	Territorial biodiversity, Tree of Life, Biomining, Astrobiology

### Growth conditions and genomic DNA preparation

The culture obtained from this reactor grew at 15 g L^−1^ Cl^−^ and exhibited sulfur oxidizing activity. Strain CLST was isolated by plating the reactors culture medium using Phytagel 1% as gelling agent. Strain CLST was grown in minimal medium (0.4 g L^−1^, (NH_4_) 2SO_4_, 0.4 g L^−1^, MgSO_4_ × 7H_2_O, 0.2 g L^−1^, K_2_HPO_4_ and 3.93 g L^−1^, CuSO_4_, pH 1.7) containing NaCl (24.7 g L^−1^). After successive subculturing (three times), DNA was isolated using High Pure Template Preparation Kit (Roche, Germany) according to the manufacturer instructions.

### Genome sequencing and assembly

The genome of 10.1601/nm.2199 strain CLST was sequenced at Beckman Coulter Genomics using 454 sequencing technology and mate pair libraries with insert sizes of ~500 bp [28]. Pyrosequencing reads were assembled de novo using Newbler (v2.0.01.14). The final draft assembly contained 389 contigs in 40 scaffolds ranging in size from 2298 bp to 409,853 bp. The total size of the genome is ~3,9 Mbp and the final assembly is based on 82 Mbp of 454 data, which provides an average 36× coverage of the genome.

### Genome annotation

Genes were predicted using Glimmer 3.02 [29] as part of the RAST annotation pipeline [30]. The tRNA and tmRNA identification was achieved using ARAGORN v1.2.36 [31] and the rRNA prediction was carried out with HMMER3 [32]. Additional gene prediction analysis and manual functional annotation was performed at the Center for Bioinformatics and Genome Biology (CBGB-FCV). The predicted CDSs were used to search the NCBI non-redundant database, UniProt, TIGRFam, Pfam, PRIAM, KEGG, COG and InterPro databases. Protein coding genes were analyzed for the presence of signal peptides using SignalP v4.1 [33] and transmembrane helices using TMHMM v2.0 [34].

## Genome properties

The draft genome contains 3,974,949 nucleotides and has an average G + C content of 48.8% (Table 3). From a total of 3941 genes, 3866 are predicted to be protein coding genes and 75 are RNA genes. The RNA genes partitioned into 1 tmRNA, 1 rRNA operon and 71 tRNAs distributed in 17 scaffolds (40% of which map to a single scaffold), suggesting the presence of an additional complete set of tRNAs as in the case of strain Licanantay [8] and 10.1601/nm.2202 type strain (10.1601/strainfinder?urlappend=%3Fid%3DATCC+23270) [35]. Predicted protein functional distributions follow highly similar profiles of other 10.1601/nm.2199 sequenced strains according to COG classification, with 36% of the genes being related to metabolism, 26% to information flux and 15% to cellular structure maintenance. A total of 43.63% of the genes were assigned a putative function while the remaining were annotated as hypotheticals. The distribution of genes in COGs functional categories is presented in Table 4.

**Table 3:** Genome statistics

Attribute	Value	% of Total^a^
Genome size (bp)	3,974,949	100.00
DNA coding (bp)	3,051,435	76.76
DNA G + C (bp)	1,939,775	48.80
DNA scaffolds	40	100.00
Total genes^b^	3941	100.00
Protein coding genes	3866	98.09
RNA genes^c^	75	1.91
Pseudo genes^d^	n.d.	n.d.
Genes in internal clusters	2118	54.78
Genes with function prediction	2468	63.38
Genes assigned to COGs	1803	46.63
Genes with Pfam domains	2634	68.13
Genes with signal peptides	292	7.55
Genes with transmembrane helices	880	22.33
CRISPR repeats	0	0.00

^a^The total is based on either the size of the genome in base pairs or the total number of genes in the annotated genome

^b^Includes tRNA, tmRNA, rRNA

^c^Includes 23S, 16S and 5S rRNA

^d^n.d.: Not determined

**Table 4:** Number of genes associated with general COG functional categories

Code	Value	%age	Description
J	165	4.27	Translation, ribosomal structure and biogenesis
A	1	0.03	RNA processing and modification
K	102	2.64	Transcription
L	121	3.13	Replication, recombination and repair
B	0	0.00	Chromatin structure and dynamics
D	31	0.80	Cell cycle control, Cell division, chromosome partitioning
V	59	1.53	Defense mechanisms
T	111	2.87	Signal transduction mechanisms
M	138	3.57	Cell wall/membrane biogenesis
N	69	1.78	Cell motility
U	46	1.19	Intracellular trafficking and secretion
O	88	2.28	Posttranslational modification, protein turnover, chaperones
C	124	3.21	Energy production and conversion
G	75	1.94	Carbohydrate transport and metabolism
E	116	3.00	Amino acid transport and metabolism
F	56	1.45	Nucleotide transport and metabolism
H	103	2.66	Coenzyme transport and metabolism
I	53	1.37	Lipid transport and metabolism
P	93	2.41	Inorganic ion transport and metabolism
Q	22	0.57	Secondary metabolites biosynthesis, transport and catabolism
R	99	2.56	General function prediction only
S	131	3.38	Function unknown
–	2063	53.36	Not in COGs

The total is based on the total number of protein coding genes in the genome

## Insights from the genome sequence

**Fig. 3:**
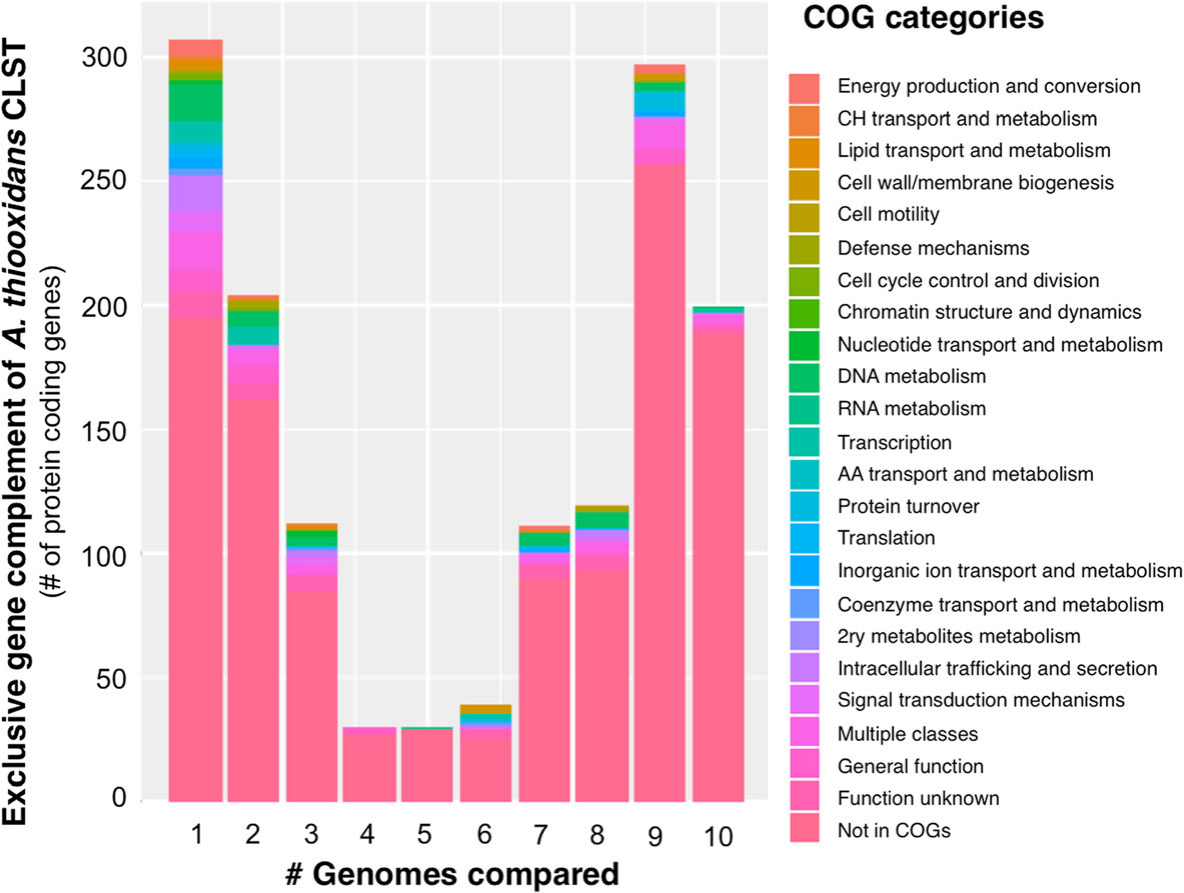
Exclusive gene complement of 10.1601/nm.2199 strain CLST relative to other strains of the species. All possible pairwise comparisons were performed. The number of exclusive genes resulting from the pairwise comparisons between the genomes of CLST and the other 10.1601/nm.2199 strains is plotted against the number of genomes compared. Genes were color coded according to their COG predicted function, as indicated in the lateral bar

10.1601/nm.2199 CLST predicted gene complement was compared against the genome of the type strain of the species (10.1601/strainfinder?urlappend=%3Fid%3DATCC+19377) and the publically available draft genomes of nine additional strains using the sequence based comparison tools of RAST [36, 37]. CLST shares 86% of its gene complement with the most similar strain in the set (Licanantay) and little over 70% with the type strain of the species (10.1601/strainfinder?urlappend=%3Fid%3DATCC+19377 ^T^). All diagnostic features of 10.1601/nm.2199 strains [1, 38, 39] are encoded in the core genome, and have been described elsewhere [7–10]. The exclusive gene complement of strain CLST encompasses 200 protein-coding genes, 95% of which are hypotheticals. An additional 1234 genes are partially shared with a subset of the strains under comparison (Fig. 3) and thus constitute the flexible gene complement. A number of these exclusive genes can be linked to osmotolerance responses, including active uptake of potassium (*kdpFABC*), synthesis of the counterion glutamate (glutamate synthase), synthesis of compatible solutes such as the aminoacid Proline (*proQ*) and possibly also polyamines (carbamoyl-phosphate synthase). Several genes involved in mitigation of other types of stress also formed part of the flexible gene pool of the CLST strain, including the ruberythrin gene cluster and a non-heme chloroperoxidase involved in oxidative stress resistance [40], copper and mercury resistance genes to withstand metal toxicity [41] and genes for the export of protective extracellular polysaccharides (*kps* system) [42]. Besides, these functions and an extensive number of hypotheticals, the CLST flexible gene complement also includes a variety of functions linked mobile genetic elements of diverse nature [43], suggesting that many of the differentiating features of CLST may have been horizontally transferred from other members of the microbial community.

## Conclusions

This work reports the first draft genome and annotation of a halotolerant acidophilic sulfur-oxidizing 10.1601/nm.2198 *(A. thioooxidans* strain CLST*),* together with its basic microbiological properties and fundamental metadata from the saline environment in northern Chile from which it was isolated. The 3.9 Mbp draft genome sequence of strain CLST is arranged in 40 high quality scaffolds, being 24% larger than the genome of the type strain and resembling in size other industrial isolates recently sequenced. It encodes 75 RNAs and 3866 predicted protein-coding genes, 43% of which were assigned putative functions. Over one third of the gene complement is flexible, being represented in few strains other than CLST. Several of the exclusive genes identified in this study can be linked to osmotolerance and other stress responses. Further study of these and other features will likely provide new insights into sodium-requiring processes in acidophilic chemolithotrophic bacteria and further understanding of the mechanisms used by acidophilic bacteria to endure high osmotic stress in natural and industrial saline environments. The release of the genome sequence of this strain improves the representation of these extreme acidophilic Gram negative bacteria in public databases and strengthens the framework for further investigation of the physiological diversity and ecological function of *A. thioooxidans*.

## Additional files

Additional file 1: Figure S1. (A, C) A. thiooxidans ATCC 19377 cell growth and growth specific rate with and without NaCl. (B, D) A. thiooxidans CLST cell growth and growth specific rate with and without NaCl. (TIFF 272 kb)

Additional file 2: Figure S2. SEM image and EDS spectrum of the precipitate obtained when A. thiooxidans was grown in a medium supplemented with CuSO4. (TIFF 206 kb)

## Abbreviations

ATCC: American Type Culture Collection
CBAR-UNC: Centro de Biotecnología “Profesor Alberto Ruiz”, Universidad Católica del Norte
DSM: Deutsche Sammlung von Mikroorganismen
FCV: Fundación Ciencia & Vida
RISC: Reduced Inorganic Sulfur Compound
