# Supplementary figures and images for: Draft genome sequence of Acidithiobacillus thiooxidans CLST isolated from the acidic hypersaline Gorbea salt flat in northern Chile
Source: Stand Genomic Sci. 2017 Dec 19;12:84. doi: 10.1186/s40793-017-0305-8 (PMC5735861; doi:10.1186/s40793-017-0305-8)

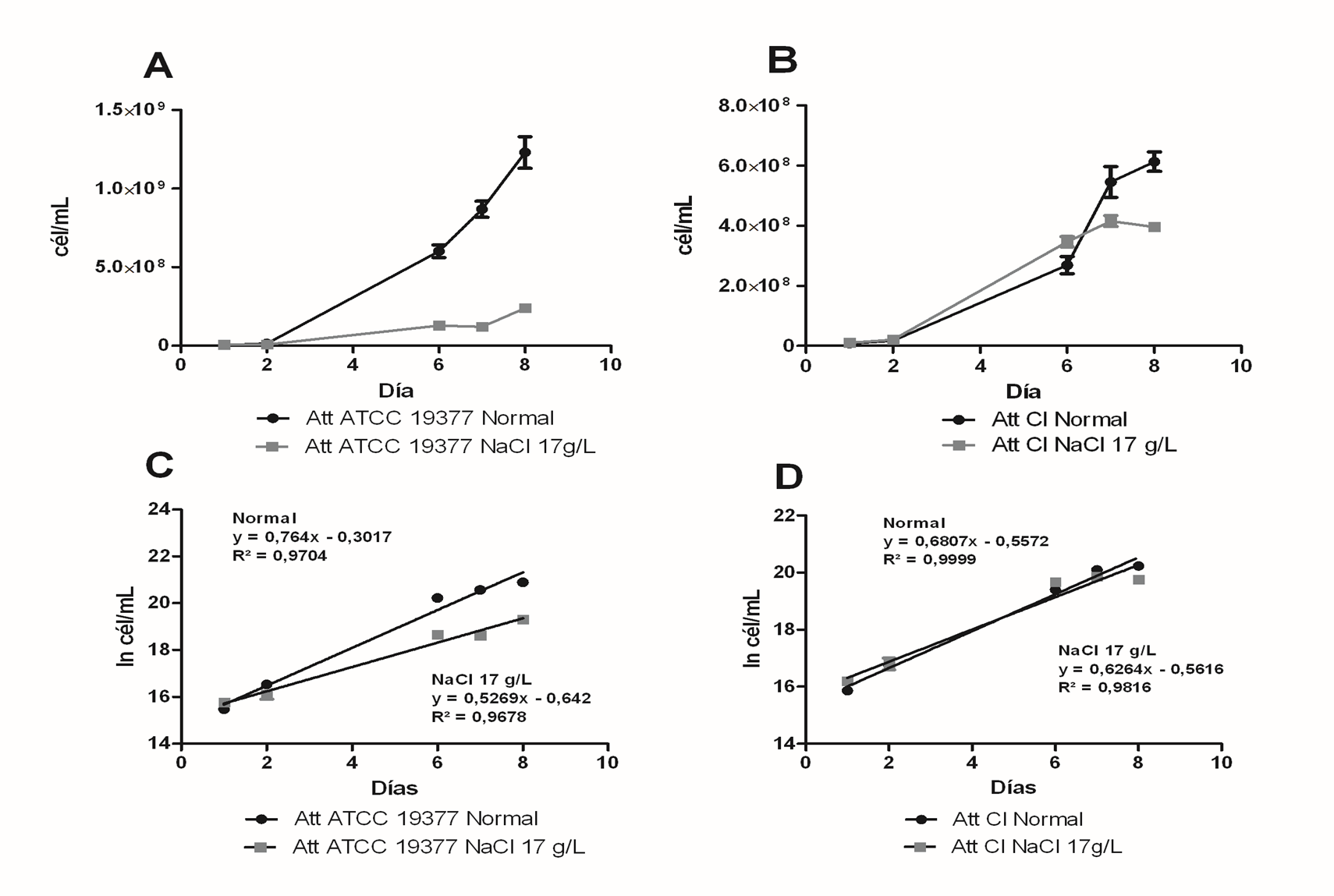

Supplement: Supplementary file 1 — (A, C) A. thiooxidans ATCC 19377 cell growth and growth specific rate with and without NaCl. (B, D) A. thiooxidans CLST cell growth and growth specific rate with and without NaCl. (TIFF 272 kb) [file 40793_2017_305_MOESM1_ESM.tif]

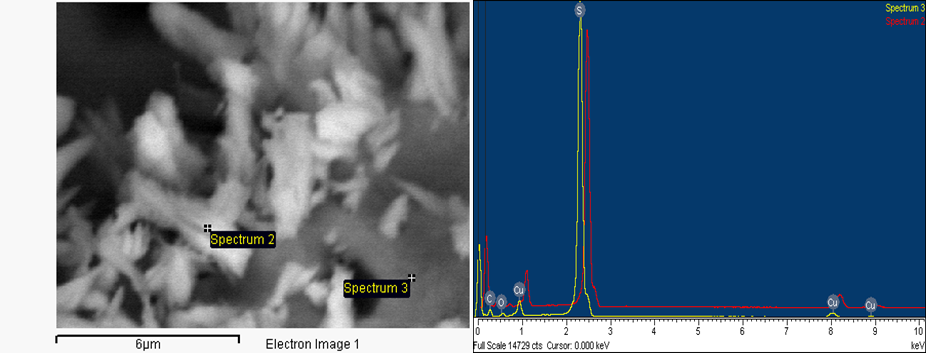

Supplement: Supplementary file 2 — SEM image and EDS spectrum of the precipitate obtained when A. thiooxidans was grown in a medium supplemented with CuSO4. (TIFF 206 kb) [file 40793_2017_305_MOESM2_ESM.tif]
